# Alcohol in Acute Disease

**Published:** 1896-10-17

**Authors:** 


### Alcohol in Acute Disease.

Speaking at the Church Congress, Sir Willoughby Wade, M.D., addressed himself to the alcohol question from the standpoint of one who had during many years studied its effects both in health and disease. He pointed out, among other things, that the fact that alcohol was sometimes injurious was no proof whatever that it could not act beneficially—any drug which was incapable of doing harm being generally equally incapable of doing good. In regard to the craving for alcohol, which was sometimes said to be set up by its use as a medicine, he thought that this did not so often follow its employment in acute disease as in those chronic ailments for the relief of which it was far mere frequently prescribed by the patients themselves, or by their friends, than by the doctor. It was, he thought, the use of alcohol for the alleviation of painful recurring conditions, such as neuralgia and asthma, and for the relief of worry and depression of spirits, that was responsible for a large proportion of the drunkenness of women and for no inconsiderable proportion of the drunkenness of men. Putting all matters of opinion on one side however, and reverting purely to experience, he insisted on the fact that in certain acute diseases conditions arose over which alcohol exercised a control such as no other drug possessed. Sometimes large doses, and even their repetition were, he said, necessary, but he had never known any craving for alcohol set up by such a mode of using it.

We think that these remarks, founded as they are on practical experience, are worthy of much consideration. The remarkable diminution which has taken place of late years in the use of alcohol in our hospitals, as was well shown in an article which we published last week, is all good so far as it indicates the cessation of a wasteful and injurious employment of a most active drug. But we must beware of the swing of the pendulum. The fact is the time has come when the action of alcohol in acute disease requires to be investigated afresh in the light of our recently acquired knowledge as to the chemical and biological pathology of these diseases. In regard to this, as Sir Willoughby Wade says, it is "futile to discuss whether or no alcohol is a food," and however interesting it may be to inquire as to its effect upon the vaso-motor system, or its influence on the activity of metabolism, these things are quite outside the real question as to the part which alcohol plays in acute diseases caused by micro-organisms. Neither the chemical constitution of such a substance as an antitoxin, so far as it is discoverable, nor its action as observed on the healthy body, could ever have given the slightest clue to its extraordinary power over disease, and in regard to alcohol it is not too much to say that attention has been far too much concentrated upon the consideration of its nutritive power. It is not by estimating its contained energy, nor by minute analysis of the end-products resulting from its digestion, that the therapeutic efficiency of a drug in acute disease can be judged of; but rather by observing its effects upon intruding organisms, and upon the production of those products of disturbed metabolism which we call antitoxins, by which their intrusion is rendered harmless. This has yet to be done in regard to alcohol, and so long as we find physicians of ripe experience declaring this substance to possess powers of controlling disease such as are possessed by no other drug, we must be chary in accepting any statements as to the uselessness of alcohol either as a food or a heat producer, as bearing in any way upon the question of its therapeutic efficiency in the treatment of acute disease.

